# From nematode identification to sustainable solution: developing tissue culture propagation for *Micranthemum* and *Cryptocoryne* ornamental aquatic plants

**DOI:** 10.1186/s12870-025-06894-z

**Published:** 2025-07-07

**Authors:** Tsung-Meng Wu, Kuang-Teng Wang, Chia-Chen Su, I-En Shih, Chia-An Liu, Yuh Tzean

**Affiliations:** 1 Department of Aquaculture, National Pingtung University of Science and Technology, Pingtung, 912301 Taiwan; 2 Department of Plant Medicine, National Pingtung University of Science and Technology, Pingtung, 912301 Taiwan; 3 Department of Plant Pathology and Microbiology, National Taiwan University, Taipei, 106319 Taiwan

**Keywords:** Plant-nematode interaction, Phytopathogen management, Explant sterilization, Micropropagation, Plant growth regulators, Substrate formulations, Sustainable horticulture practices

## Abstract

Ornamental aquarium plants, particularly the genera *Micranthemum* and *Cryptocoryne*, are prized for enhancing aquascapes’ aesthetics. However, they face challenges in propagation due to susceptibility to pests and complex cultivation requirements. This study aimed to develop in vitro propagation techniques for *Micranthemum* and *Cryptocoryne* spp. By using a 0.5% sodium hypochlorite solution for disinfection, we developed effective methods for establishing nematode-free cultures. For *Micranthemum* sp. ‘Monte Carlo’, the optimal condition for adventitious bud proliferation was 0.1 mg/L 1-Naphthaleneacetic acid (NAA), while *M. glomeratus* showed optimal proliferation with plant growth regulator (PGR)-free media. *Cryptocoryne* sp. ‘Flamingo’ exhibited optimal proliferation when treated with 6-benzylaminopurine (BAP) at 4.0 or 6.0 mg/L combined with 0.1 mg/L NAA. For root formation, both *Micranthemum* sp. ‘Monte Carlo’ and *M. glomeratus* performed best in PGR-free conditions, while *Cryptocoryne* sp. ‘Flamingo’ achieved the highest root formation with 0.4 mg/L BAP and NAA-free media. During acclimatization, all three species showed a 100% survival rate across all tested substrates—silica sand, a mixture of aqua soil and silica sand, and exclusively aqua soil, with the latter outperforming others in promoting growth. This study presents robust in vitro propagation protocols that not only enhance the proliferation and growth of popular ornamental aquarium plants but also promise to mitigate the propagation challenges they face, contributing towards sustainable ornamental horticulture and reinforcing the ecological and aesthetic value of aquascaping.

## Key Message

This study presents effective tissue culture for *Micranthemum* and *Cryptocoryne* spp., offering sustainable solutions to enhance propagation and manage phytopathogens in ornamental aquatic plant cultivation.

## Introduction

Ornamental aquatic plants encompass a diverse array of species notable for their aesthetic attributes, including unique shapes, colors, and diverse sizes. These plants are integral to the embellishment of aquariums and water gardens, playing an important role in the enhancement of aquatic landscapes [1, 2]. Such plants are typically cultivated externally before transplantation into aquatic settings, contributing significantly to the expansion of the aquarium and water garden industries worldwide [3]. This burgeoning interest has been further amplified by the art of aquascaping, which involves the intricate arrangement of aquatic plants to create visually appealing underwater landscapes. Aquascaping not only demands a broad spectrum of plant species for various design needs but also emphasizes species selection based on their growth habits and suitability for different areas within the aquarium, from foreground and middle ground to background positions [4].

The genus *Micranthemum*, synonymous with *Hemianthus* and classified under the Linderniaceae family, originates from the Eastern United States to tropical and subtropical regions of America. This genus encompasses 13 accepted species that are highly valued for their ornamental appeal in aquatic settings [5–8]. Among these, *Micranthemum* sp. ‘Monte Carlo’ and *Micranthemum glomeratum* (*Hemianthus glomeratum*), commonly referred to as pearl grass, are especially esteemed for their capacity to create dense, carpet-like effects in freshwater aquariums [7]. This ability significantly enhances both the aesthetic value and perceived spatial dimensions of aquatic environments. Prior research has demonstrated successful propagation of *H. callitrichoides* ‘Cuba’ using MS medium supplemented with 0.50 mg/L 6-benzylaminopurine (BAP), which significantly improved shoot clump diameter, area, and weight [7]. However, high sucrose concentrations (9%) were found to inhibit growth and induce abnormalities. Furthermore, *Hemianthus callitrichoides* showed optimal clump diameter (5.53 cm) and enhanced growth on MS No:3B medium supplemented with 20 g/L sucrose and 1 g/L agar, without the addition of plant growth regulators (PGRs) [9]. Another study showed that for *H. micranthemoides*, the highest shoot clump diameter and area was obtained in MS medium containing 0.075 mg/L 1-phenyl-3-1,2,3-thiadiazol-5-yl urea (TDZ) and 0.1 mg/L naphteneacetic acid (NAA) [8]. These findings highlight the importance of optimizing plant growth regulators (PGRs) and medium composition for the successful micropropagation of *Micranthemum* spp.

Furthermore, the genus *Cryptocoryne*, belonging to the Araceae family, consists of approximately 50–60 aquatic monocot species that are predominantly native to Southeast Asia and Indonesia. These species are adaptable to both submerged or emerged conditions, making them versatile additions to aquatic landscapes [4, 10–18]. Among these, *Cryptocoryne* sp. ‘Flamingo’ is distinguished by its unique pink foliage. However, it presents a cultivation challenge due to its slow rhizome growth and limited seed production, which restricts its availability [13, 14, 19]. Previous research on *Cryptocoryne* sp. has shown that shoot proliferation can vary depending on the culture medium and growth regulators. *C. wendtii* and *C. becketti* produced more shoots in liquid cultures compared to solid media, with 9.4 and 8.8 shoots per explant, respectively, when MS medium was supplemented with 0.5 mg/L BA and 0.2 mg/L IBA [16]. Similarly, *C. wendtii* showed a sevenfold increase in shoot production on MS medium with 20 µM BA alone [13], *and C. lucens* produced 7.7 shoots per explant in LS medium with 20 µM BA and 0.5 µM NAA [14]. Additionally, the highest number of shoots for *C. wendtii* (7.21 shoots per explant) was observed on MS medium with 4.0 mg/L BA and 1.0 mg/L IBA [20], while 100% of explants cultured on MS medium with 3.0 mg/L BAP showed successful shoot proliferation, with up to 16.2 shoots per explant after 60 days and 72.4 leaves per explant [21].

The propagation and cultivation of these aquatic plants are pivotal for both ecological balance and the ornamental plant industry. Traditional propagation methods have relied on both sexual (seed-based) and asexual (vegetative) mechanisms. Yet, aquatic plants, particularly those grown in natural or horticultural settings, are vulnerable to a range of phytopathogens and pests and can cause extensive damage, significantly hindering international trade due to stringent regulations against their spread [22]. To address these challenges, the application of tissue culture techniques is an effective alternative for the rapid, true-to-type propagation of various plant species, thus facilitating large-scale micropropagation of high-quality clones in a relatively short timeframe [2, 23–25]. However, while there have been substantial advancements in the micropropagation protocols for terrestrial plants over recent decades, the in vitro culture of aquatic plant species such as *Micranthemum* sp. ‘Monte Carlo’, *M. glomeratus*, and *Cryptocoryne* sp. ‘Flamingo’ remains to be further developed despite their commercial significance in the aquarium industry.

In this study, we observed nematode infestations among aquatic plant species: *Micranthemum* sp. ‘Monte Carlo’, *M. glomeratus*, and *Cryptocoryne* sp. ‘Flamingo’. Consequently, we developed an efficient method for the disinfection of explants suitable for in vitro culture, optimized the utilization of plant growth regulators (PGRs) to enhance plantlet regeneration and multiplication rates, and determined the ideal aquarium substrates for facilitating the acclimation of plantlets during the transition from in vitro to ex vitro conditions. The refinement in the in vitro and ex vitro culture parameters facilitated the establishment of a comprehensive protocol for the efficient mass propagation of these specified species. The application of this research would not only increase the availability of these desirable aquatic plants but also ameliorate the challenges posed by nematode infestations, thereby contributing to the sustainable development of the ornamental aquatic plant sector.

## Materials and methods

### Plant materials and microscopic observations of nematodes

The visually healthy aquatic plant species of *Micranthemum* sp.‘Monte Carlo’, *Cryptocoryne* sp.‘Flamingo’, and *M. glomeratus* were purchased from a local aquarium stores in Taipei, Taiwan. The plants were maintained in aquariums until use in this study. For microscopic evaluation, nematodes were isolated from the plants using a modified Baermann funnel method [22].

### Acquisition of aseptic explants from aquatic plants

Shoot tips (~ 1.0 cm long) of *Cryptocoryne* sp. ‘Flamingo’ with their roots and leaves removed were used as the explant. Nodal segments (~ 1 cm long) of *Micranthemum* sp. ‘Monte carlo’ and *M. glomeratus* were used as the explant. The explants were washed in running tap water for 1 h, followed by rinsing in sterilized double distilled water. Thereafter, they were surface-sterilized using commercial bleach (containing 6% sodium hypochlorite, Bailan, Taiwan) diluted into 0.5%, 1%, 2% and 3% sodium hypochlorite solutions for 10, 20 and 30 min and supplemented with Tween 20. Finally, they were rinsed at least 6 times with sterile distilled water, each rinse performed for 3–5 s, and blotted on sterilized tissue paper. Thereafter, the *Cryptocoryne* sp. ‘Flamingo’ explants were cultured in solid MS medium [26] containing 3% (w/v) sucrose and 0.5% (w/v) Agar A (Bio Basic, Markham, Canada). For *Micranthemum* spp., the explants were cultured in solid ½ MS medium [26] containing 3% (w/v) sucrose and 0.5% (w/v) Agar A (Bio Basic, Markham, Canada). The pH of the medium was adjusted to 5.7, and the medium was dispensed as 20 mL aliquots into Petri dishes after autoclaving. Each disinfection treatment consisted of four replicated Petri dishes, with ten explants per replicate. Explants were cultured in a growth chamber (F-740, HiPoint, Kaohsiung, Taiwan) at 25 ± 1 °C with a light intensity of 50 µmol m^–2^ s^–1^ provided by cool white fluorescent lights, a 12-h photoperiod. Visible contamination of the explants was recorded after in vitro culture for 4 weeks. The percentage of contaminated explants was calculated as the number of contaminated explants divided by the total number of explants multiply by 100.

In order to obtain enough numbers of explants for the following studies, the axenic explants of three species were subcultured in solid MS medium supplemented with 0.5 mg/L 6-benzylaminopurine (BAP) according to the previous studies [7, 20].

### Effects of plant growth regulators on shoot proliferation and root formation

To evaluate the optimum concentration of BAP and 1-Naphthaleneacetic acid (NAA) for shoot proliferation of three species, the aseptic explants were inoculated into Petri dish containing solid MS medium for *Cryptocoryne* sp. and 1/2 MS medium for *Micranthemum* spp. supplemented with different concentrations of BA (0, 2, 4, or 6 mg/L) and NAA (0, 0.1, or 0.2 mg/L) for 4 weeks. For root formation experiment, the aseptic explants, which had undergone multiple subculturing cycles (subcultured every four weeks), were transferred directly from the optimized shoot proliferation medium to solid MS medium supplemented with different concentrations of BA (0.2, 0.4, or 0.6 mg/L) and NAA (0.1, 0.5, or 1.0 mg/L) for 4 weeks. Each treatment consisted of four replicated Petri dishes, with five explants per replicate. The entire experiment was repeated at least three times. Culture conditions in the growth chambers were as described above. The rate of shoot proliferation and root formation was calculated as the number of explants with shoot proliferation or clump formation and root formation divided by the total number of explants, respectively. The parameters used in the study were number of shoots and roots, average shoot height and root length, average clump diameter and weight.

### Impact of substrate compositions on the acclimatization of aquatic plants post tissue culture

The choice of in vitro plantlets for acclimation depends on the type of plant. Nodal segments with apical buds (~ 2 cm long) of *Micranthemum* sp. ‘Monte Carlo’ and *M. glomeratus* were used. Plantlets of *Cryptocoryne* sp. ‘Flamingo’ with shoot heights ranging from 4 to 5 cm, roots numbering between 4 and 6 roots, and leaves ranging from 3 to 6 leaves were utilized. The roots of these plantlets were then cut and shortened to a size of 0.5 cm prior to *ex vitro* acclimatization. Plantlets were carefully cleaned and removed all the residual media were removed to prevent pathogenic contamination. The cleaned plantlets were then transplanted into Aqua soil (ADA, Japan), silica sand (Aqua plus, Taiwan) and mixture of Aqua soil and silica sand (1:1) containing in aquariums (12 × 12 × 18 cm) saturated with water and covered with a transparent plastic cover to maintain a high relative humidity. Each treatment consisted of four replicated aquariums, with 20 plantlets per replicate. The percentage of surviving plantlets was recorded after 4 weeks of acclimatization.

### Statistical analysis

Experimental results were subjected to a one-way Analysis of Variance (ANOVA), followed by post hoc comparisons using Duncan’s multiple range test to ascertain significant variances among treatment groups, setting the level of significance at *P* < 0.05. Statistical evaluations were performed utilizing the SPSS software, version 20.0. The findings are delineated as mean ± standard deviation (SD).

## Results

### Microscopic evaluation of nematode observations

**Fig. 1:**
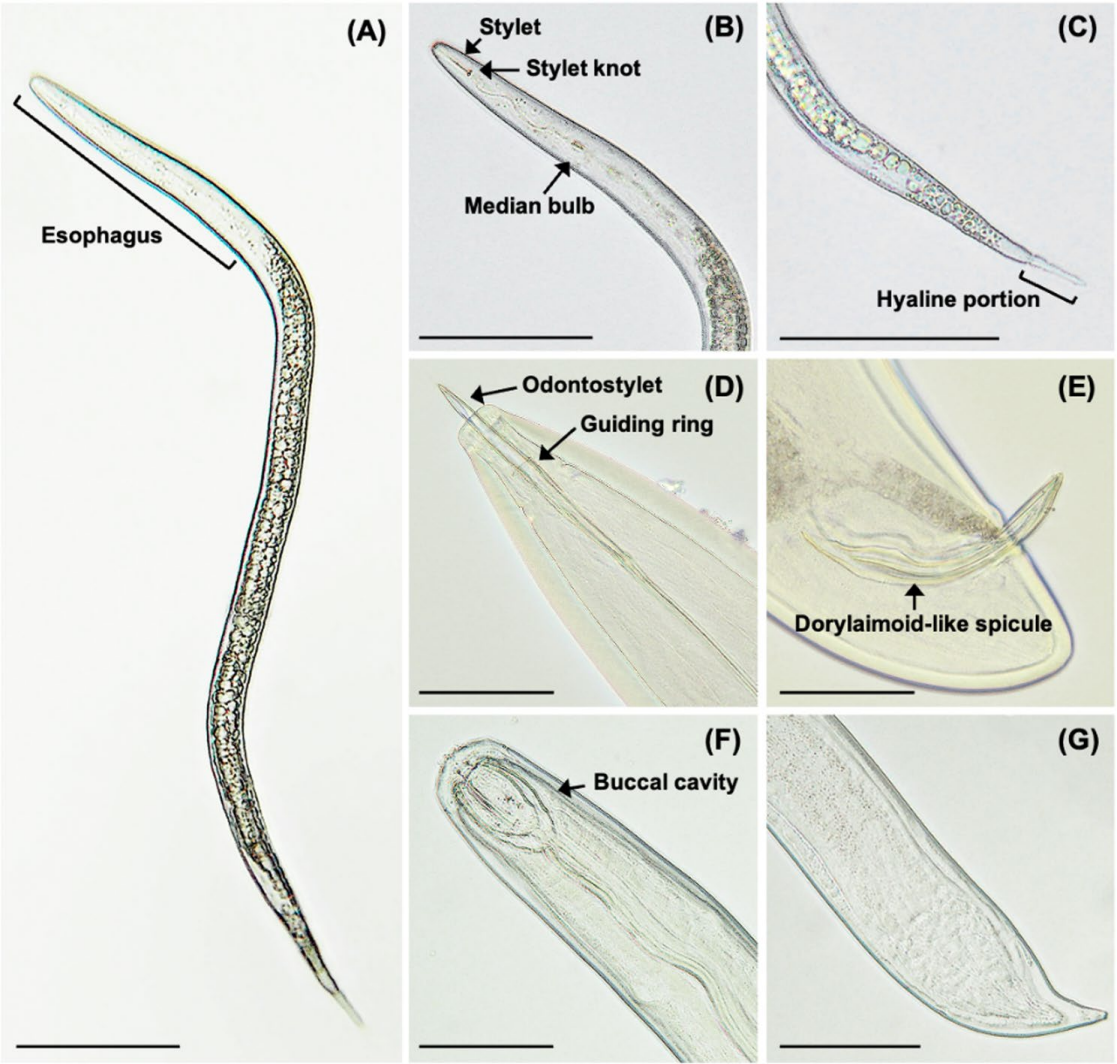
Morphological traits of nematodes isolated in the study. **A** Micrograph depicting a second-stage juvenile of a plant-parasitic nematode with an elongated esophagus constituting approximately one-third of the body length. **B** Displaying a non-distinct lip region, a slender and elongated stylet, and an ovoid median bulb. **C** Tail characterized by a transparent hyaline region and irregular tapering. **D** Members of the Dorylaimidae family with an encircling guiding ring around the odontostyle. **E** Male nematodes featuring a dorylaimoid-like spicule within the Dorylaimidae family. **F** Large buccal cavity representative of a predatory nematode. **G** Digitate-like tail structure. Scale bar = 100 μm

During our investigation, microscopic evaluation of nematodes extracted from the aquatic plant species *Micranthemum* sp. ‘Monte Carlo’, *M. glomeratus*, and *Cryptocoryne* sp. ‘Flamingo’ revealed nematodes with distinct morphological traits consistent with both plant-parasitic and free-living feeding strategies (Fig. 1). Notably, a subset of nematodes demonstrated an elongated esophagus (Fig. 1A) coupled with pronounced stylet and stylet knobs (Fig. 1B), characteristic of plant-parasitic nematodes. Furthermore, a distinctive tail with a hyaline region narrowing irregularly was also observed (Fig. 1C). Conversely, nematodes belonging to the family Dorylaimidae were identified by the presence of an odontostyle encircled by a guiding ring (Fig. 1D), typically indicative of a predatory lifestyle. These nematodes are equipped with robust feeding apparatuses, enabling them to prey on other microorganisms or nematodes. Additionally, the observation of dorylaimoid-like spicules (Fig. 1E) indicates the presence of male nematodes. Nematodes encompassing large buccal cavity (Fig. 1F) with harboring a digitate-like tail (Fig. 1G) were also observed. The presence of phytoparasitic and free-living nematodes reflects a multifaceted nematode community associated with aquatic plant species *Micranthemum* sp. ‘Monte Carlo’, *M. glomeratus*, and *Cryptocoryne* sp. ‘Flamingo’.

### Acquisition of aseptic explants from aquatic plants

To obtain sterile plants, we first evaluated the effects of sodium hypochlorite solution concentration and plant immersion time on obtaining sterile explants of three aquatic plants (Table 1). The optimal disinfection condition for *Micranthemum* sp. ‘Monte Carlo’ was identified as immersing in a 0.5% sodium hypochlorite solution for 30 min, achieving a 47.5 ± 12.6% aseptic rate, followed by a 1.0% solution for 20 min with a 35.0 ± 12.9% aseptic rate. Similarly, *M. glomeratus* exhibited the highest aseptic rate of 65.0 ± 15.1% under the same conditions (i.e. 0.5% for 30 min), followed by 27.5 ± 9.6% for a 1.0% solution for 10 min. *Cryptocoryne* sp. ‘Flamingo’ demonstrated optimal aseptic rate from immersion in a 0.5% solution for 30 min with 50.0 ± 8.2%, followed by 40.0 ± 8.2% and 32.5 ± 9.6% soaking in 1.0% solutions soaked for 20 and 30 min, respectively (Table 1).

**Table 1:** Effect of sodium hypochlorite solution concentration and immersion time on the acquisition of aseptic explants

NaOCl (%)	Duration time (min)	% Aseptic explants of Micranthemum sp. ‘Monte carlo’ *	% Aseptic explants of Micranthemum glomeratus*	% Aseptic explants of Cryptocoryne sp. ‘Flamingo’ *
0.5	10.0	0.0 ± 0.0^d^	0.0 ± 0.0^d^	0.0 ± 0.0^d^
	20.0	12.5 ± 9.6^bc^	19.0 ± 14.5^bc^	10.0 ± 8.2^c^
	30.0	47.5 ± 12.6^a^	65.0 ± 15.1^a^	50.0 ± 8.2^a^
1.0	10.0	25.5 ± 5.8^b^	27.5 ± 9.6^b^	15.0 ± 12.9^bc^
	20.0	35.0 ± 12.9^ab^	20.0 ± 8.2^bc^	40.0 ± 8.2^ab^
	30.0	0.0 ± 0.0^d^	0.0 ± 0.0^d^	32.5 ± 9.6^b^
2.0	10.0	5.0 ± 5.8^cd^	2.5 ± 5.0^c^	0.0 ± 0.0^d^
	20.0	0.0 ± 0.0^d^	0.0 ± 0.0^d^	7.5 ± 5.0^c^
	30.0	0.0 ± 0.0^d^	0.0 ± 0.0^d^	0.0 ± 0.0^d^
3.0	10.0	0.0 ± 0.0^d^	0.0 ± 0.0^d^	0.0 ± 0.0^d^
	20.0	0.0 ± 0.0^d^	0.0 ± 0.0^d^	0.0 ± 0.0^d^
	30.0	0.0 ± 0.0^d^	0.0 ± 0.0^d^	0.0 ± 0.0^d^

*Data are presented as mean ± standard deviation (*n* = 4). Different letters within the same column indicate significant differences among treatments (Duncan’s new multiple range test, *p* < 0.05)

### The effect of plant growth regulators on the proliferation of adventitious buds in aquatic plants

**Fig. 2:**
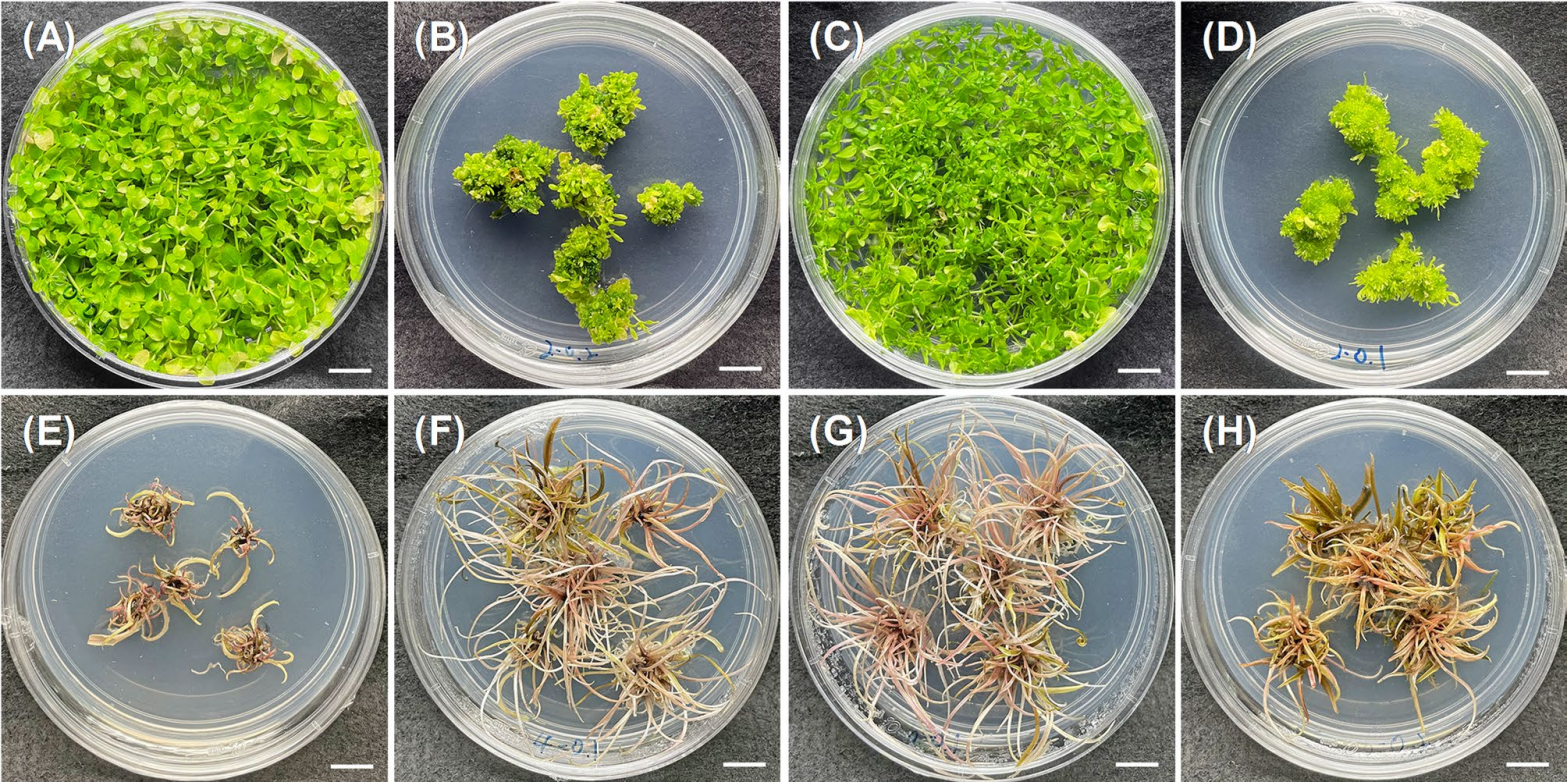
Influence of plant growth regulators on adventitious bud proliferation in *Micranthemum* sp. ‘Monte Carlo’ (**A**-**B**), *M. glomeratus* (**C**-**D**) and *Cryptocoryne* sp. ‘Flamingo’ (**E**-**H**). A is 0.1 mg/L NAA-containing ½ MS medium; **B** is 2.0 mg/L BAP and 0.2 mg/L NAA containing ½MS medium; **C** is PGR-free ½MS medium; **D** is 2.0 mg/L BAP and 0.1 mg/L NAA containing ½MS medium; **E** is PGR-free MS medium; **F** is 4.0 mg/L BAP and 0.1 mg/L NAA containing MS medium; **G** is 6.0 mg/L BAP and 0.1 mg/L NAA containing MS medium; **H** is 6.0 mg/L BAP and 0.2 mg/L NAA containing MS medium. Scale bar = 1.0 cm

The impact of plant growth regulators (PGRs) on the proliferation of adventitious buds in *Micranthemum* sp. ‘Monte Carlo’ was systematically examined (Table 2). In groups devoid of BAP, *Micranthemum* sp. ‘Monte Carlo’ exhibited vegetative growth characterized by the extension of stolon (Fig. 2A), culminating in the formation of bud clusters with dimensions and masses spanning 3.98 ± 0.35 to 4.28 ± 0.33 cm in diameter and 2.26 ± 0.05 to 2.7 ± 0.07 g, respectively. Conversely, the incorporation of BAP across all tested concentrations led to a morphological shift in the explants, promoting the differentiation into an abundance of diminutive adventitious bud clusters. These clusters were notably smaller and lighter, with diameters ranging from 1.84 ± 0.24 to 2.20 ± 0.20 cm and weights from 0.60 ± 0.01 to 0.91 ± 0.06 g, indicating a significant deviation from the BAP-free groups (Table 2; Fig. 2B). Thus, the largest clump of *Micranthemum* sp. ‘Monte Carlo’ was obtained on basal medium supplemented with 0.1 mg/L Naphthaleneacetic acid (NAA).

**Table 2:** Influence of plant growth regulators on adventitious bud proliferation in *Micranthemum* sp. ‘Monte carlo’, *M. glomeratus*, and *Cryptocoryne* sp. ‘Flamingo’

BAP (mg/L)	NAA (mg/L)	% Clumps/ Adventitious buds formation	Micranthemum sp.‘Monte carlo’		Hemianthus micranthemoide		Cryptocoryne sp.‘Flamingo’	
			Clump diameter (cm)*	Clump weight (g)*	Clump diameter (cm)*	Clump weight (g)*	Number of shoots per explant	Average length of shoots (cm)
0.0	0.0	100.0	4.28 ± 0.33^a^	2.51 ± 0.05^b^	4.37 ± 0.34^a^	2.04 ± 0.03^a^	6.4 ± 1.0^e^	1.82 ± 0.11^d^
	0.1	100.0	4.24 ± 0.17^a^	2.70 ± 0.07^a^	4.26 ± 0.24^a^	1.96 ± 0.04^b^	9.2 ± 2.6^de^	1.24 ± 0.10^ef^
	0.2	100.0	3.98 ± 0.35^a^	2.26 ± 0.05^c^	4.04 ± 0.29^a^	1.70 ± 0.07^c^	4.4 ± 1.2^e^	0.72 ± 0.07^g^
2.0	0.0	100.0	2.00 ± 0.22^b^	0.83 ± 0.01^ef^	2.30 ± 0.21^c^	0.87 ± 0.05^d^	15.8 ± 4.8^cd^	2.38 ± 0.21^c^
	0.1	100.0	1.84 ± 0.24^b^	0.68 ± 0.05^h^	2.57 ± 0.51^b^	0.87 ± 0.02^d^	17.6 ± 5.6^bcd^	2.72 ± 0.33^c^
	0.2	100.0	1.94 ± 0.20^b^	0.91 ± 0.06^d^	1.82 ± 0.17^c^	0.93 ± 0.01^d^	22.4 ± 4.6^abc^	2.48 ± 0.28^c^
4.0	0.0	100.0	1.94 ± 0.17^b^	0.68 ± 0.02^h^	2.20 ± 0.30^c^	0.71 ± 0.03^ef^	22.6 ± 9.1^abc^	1.16 ± 0.16^f^
	0.1	100.0	2.04 ± 0.16^b^	0.72 ± 0.03^gh^	2.00 ± 0.26^c^	0.61 ± 0.01^g^	25.0 ± 6.2^ab^	3.38 ± 0.28^b^
	0.2	100.0	2.20 ± 0.20^b^	0.89 ± 0.03^de^	2.14 ± 0.37^c^	0.76 ± 0.04^e^	15.6 ± 5.7^cd^	1.62 ± 0.20^de^
6.0	0.0	100.0	1.92 ± 0.17^b^	0.78 ± 0.01^fg^	1.78 ± 0.07^c^	0.66 ± 0.02^fg^	16.8 ± 5.2^bcd^	4.90 ± 0.71^a^
	0.1	100.0	1.98 ± 0.24^b^	0.66 ± 0.03^hi^	1.82 ± 0.09^c^	0.63 ± 0.03^g^	28.2 ± 2.8^a^	3.44 ± 0.18^b^
	0.2	100.0	2.02 ± 0.20^b^	0.60 ± 0.01^i^	2.00 ± 0.24^c^	0.87 ± 0.02^d^	27.2 ± 8.4^a^	2.68 ± 0.22^c^

*Data are presented as mean ± standard deviation (*n* = 4). Different letters within the same column indicate significant differences among treatments (Duncan’s new multiple range test, *p* < 0.05)

Similarly, observations were recorded for *M. glomeratus* (Table 2; Fig. 2C), which also displayed enhanced vegetative propagation via stolon in the absence of BAP, forming bud clusters with diameters between 4.04 ± 0.29 and 4.37 ± 0.34 cm, and weights from 1.70 ± 0.07 to 2.04 ± 0.03 g. The addition of BAP induced a proliferation of finer adventitious buds across all treated groups, resulting in clusters with reduced dimensions and mass (1.78 ± 0.07 to 2.57 ± 0.51 cm in diameter and 0.61 ± 0.01 to 0.93 ± 0.01 g in weight) compared to the non-BAP treatments (Table 2; Fig. 2D). According to the results above, the largest clump of *M. glomeratus* was obtained on basal medium without any plant growth regulators.

For *Cryptocoryne* sp. ‘Flamingo’, an increase in the number of adventitious buds was observed with elevated BAP concentrations, although the response to NAA varied (Table 2; Fig. 2E-H). The treatment combining 6.0 mg/L BAP with NAA (0.1 and 0.2 mg/L) proved most efficacious, catalyzing the development of 28.2 ± 2.8 and 27.2 ± 8.4 adventitious buds per explant, followed closely by 4.0 mg/L BAP with 0.1 mg/L NAA, which facilitated the emergence of 25.0 ± 6.2 buds.

### The impact of plant growth regulators on root induction in aquatic plants

**Fig. 3:**
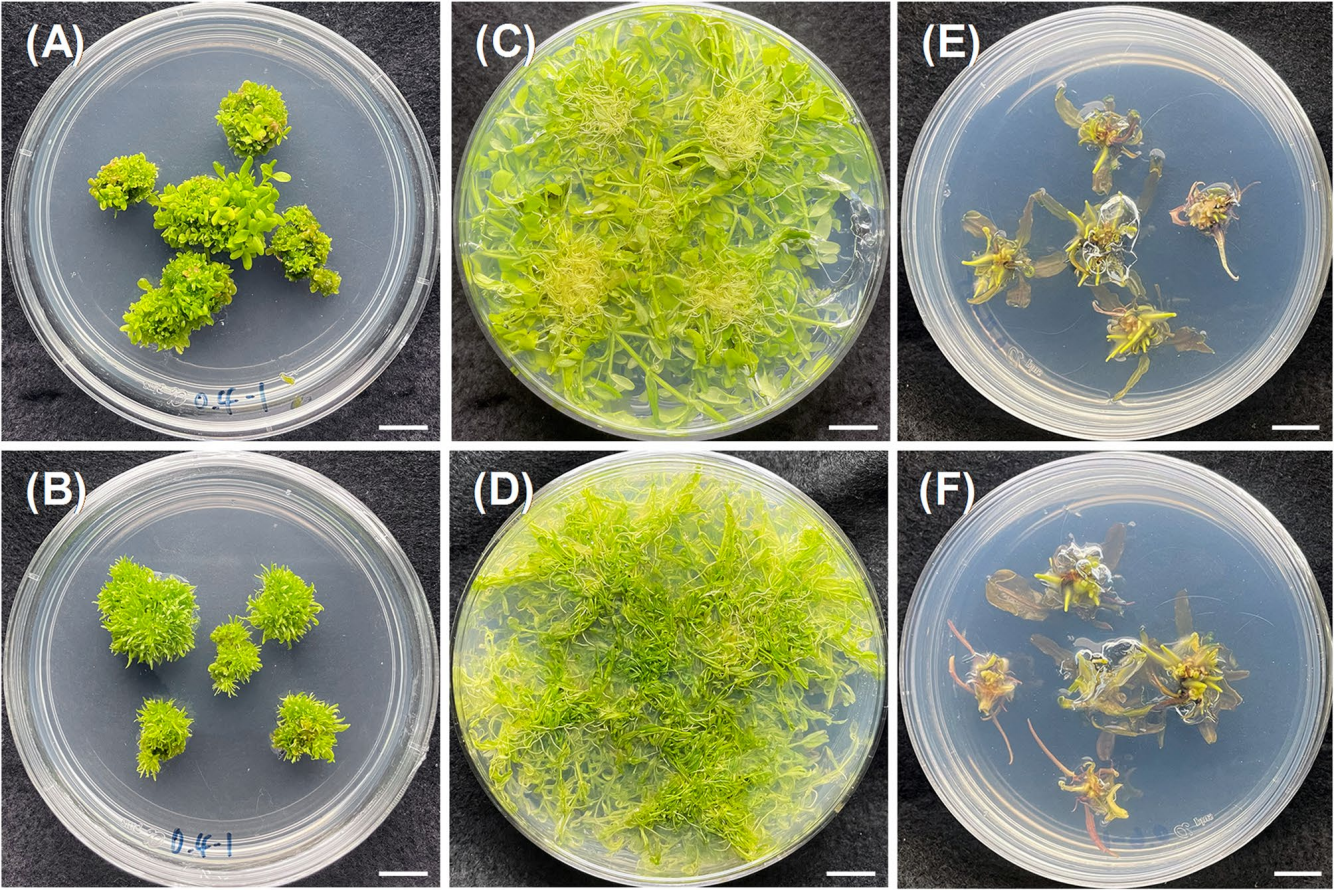
Effect of plant growth regulators on root formation in *Micranthemum* sp. ‘Monte Carlo’ (**A**, **C**), *M. glomeratus* (**B**, **D**) and *Cryptocoryne* sp. ‘Flamingo’ (**E**-**F**). A and B contain 0.4 mg/L BAP and 1.0 mg/L NAA containing ½MS medium; **C** and **D** are PGR-free ½MS medium; **E** is 0.4 mg/L BAP and 0.5 mg/L NAA containing MS medium; **F** is 0.6 mg/L BAP and 1.0 mg/L NAA containing MS medium; Scale bar = 1.0 cm

We further investigated the effects of PGRs on the root induction of *Micranthemum* sp. ‘Monte Carlo’ and *M. glomeratus* (Table 3). Across all experimental conditions, no successful root induction was observed in these species. The external appearance of the explants and the resulting proliferation of small, undifferentiated bud clusters resembled those seen during the adventitious bud induction phase (Fig. 3A and B). When these clusters of minute buds were subsequently cultured in an environment devoid of PGRs, normal growth resumed, and differentiation into roots was noted (Fig. 3C and D).

**Table 3:** Effect of plant growth regulators on root formation in *Micranthemum* sp. ‘Monte carlo’, *M. glomeratus*, and *Cryptocoryne* sp. ‘Flamingo’

BAP (mg/L)	NAA (mg/L)	Micranthemum sp.‘Monte carlo’		Hemianthus micranthemoide		Cryptocoryne sp.‘Flamingo’		
		% Adventitious roots formation	Clump diameter (cm)*	% Adventitious roots formation	Clump diameter (cm)*	% Adventitious roots formation	Average No. of adventitious roots per explant	Average length of adventitious roots (cm)
0.2	0.1	0.0	1.98 ± 0.87	0.0	2.04 ± 0.30^ab^	100.0	3.6 ± 1.3^cd^	0.58 ± 0.21
	0.5	0.0	2.54 ± 0.48	0.0	2.36 ± 0.48^a^	100.0	2.2 ± 0.7^d^	0.64 ± 0.10
	1.0	0.0	1.92 ± 0.40	0.0	2.06 ± 0.23^ab^	100.0	4.0 ± 2.8^cd^	0.54 ± 0.10
0.4	0.1	0.0	1.94 ± 0.13	0.0	1.64 ± 0.13^bcd^	100.0	3.8 ± 1.2^cd^	1.42 ± 1.79
	0.5	0.0	1.68 ± 0.33	0.0	1.46 ± 0.16^cd^	100.0	10.2 ± 2.9^a^	1.32 ± 0.11
	1.0	0.0	1.80 ± 0.26	0.0	1.82 ± 0.26^bc^	100.0	4.8 ± 2.5^cd^	0.38 ± 0.07
0.6	0.1	0.0	1.76 ± 0.04	0.0	1.70 ± 0.20^bcd^	100.0	3.8 ± 1.2^cd^	1.16 ± 0.17
	0.5	0.0	1.96 ± 0.14	0.0	1.98 ± 0.42^ab^	100.0	5.8 ± 2.7^bc^	0.70 ± 0.22
	1.0	0.0	1.68 ± 0.11	0.0	1.32 ± 0.14^d^	100.0	8.2 ± 1.7^ab^	0.80 ± 0.16

*Data are presented as mean ± standard deviation (*n* = 4). Different letters within the same column indicate significant differences among treatments (Duncan’s new multiple range test, *p* < 0.05)

Furthermore, we evaluated the influence of PGRs on root induction in *Cryptocoryne* sp. ‘Flamingo’ (Table 3). Root differentiation was observed in all treatments, with the combination of 0.4 mg/L BAP and 0.5 mg/L NAA resulting in the highest number of roots per explant, averaging 10.2 ± 2.9 roots. This was followed by the combination of 0.6 mg/L BAP with 1.0 mg/L NAA, which yielded an average number of roots at 8.2 ± 1.7 roots per explant. Notably, there were no significant differences in root length across the treatment groups. Figure 3E and F illustrates the morphological characteristics of roots in *Cryptocoryne* sp. ‘Flamingo’ under these conditions, showcasing a stout and short appearance ideal for direct planting in soil without the need for further trimming.

### Impact of substrate compositions on the acclimatization of aquatic plants post tissue culture

**Fig. 4:**
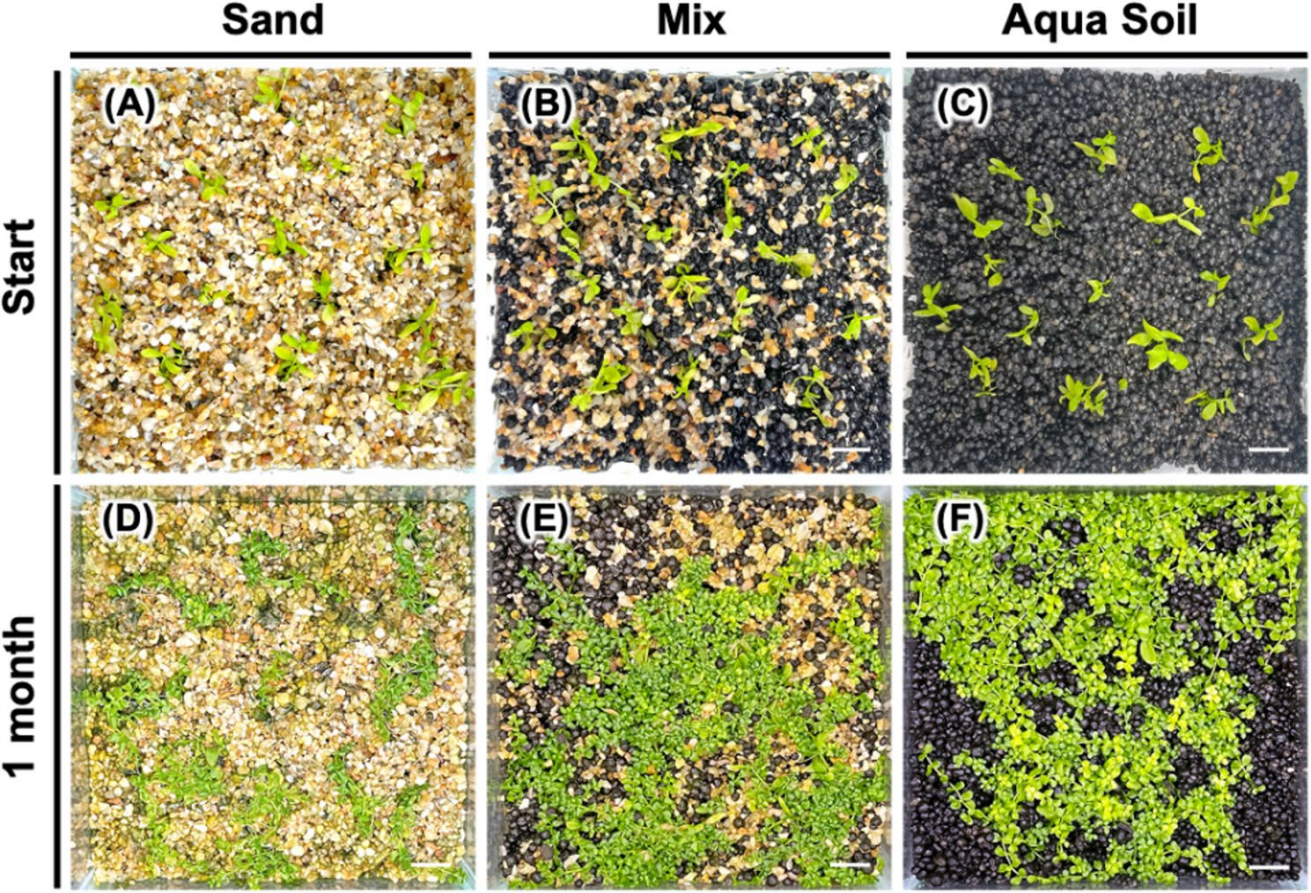
Effect of substrate composition on acclimatization of *Micranthemum* sp. ‘Monte Carlo’. Plantlets were transplanted into US fine sand (**A**), mixed substrate (**B**), and black soil (**C**), and observed after one month (**D**, **E**, **F**)

**Fig. 5:**
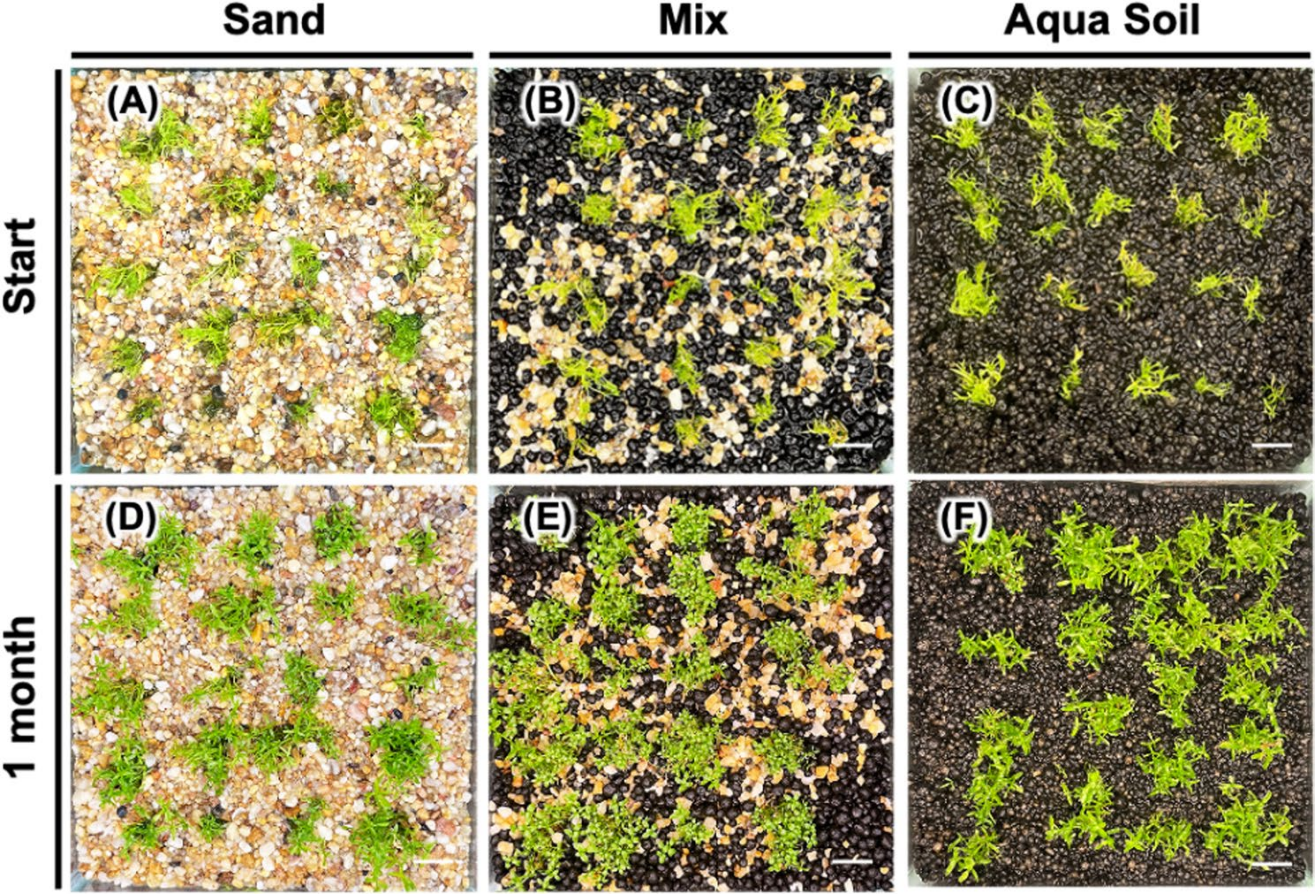
Effect of substrate composition on acclimatization of *Micranthemumglomeratus*. Plantlets were transplanted into US fine sand (**A**), mixed substrate (**B**), and black soil (**C**), and observed after one month (**D**, **E**, **F**)

**Fig. 6:**
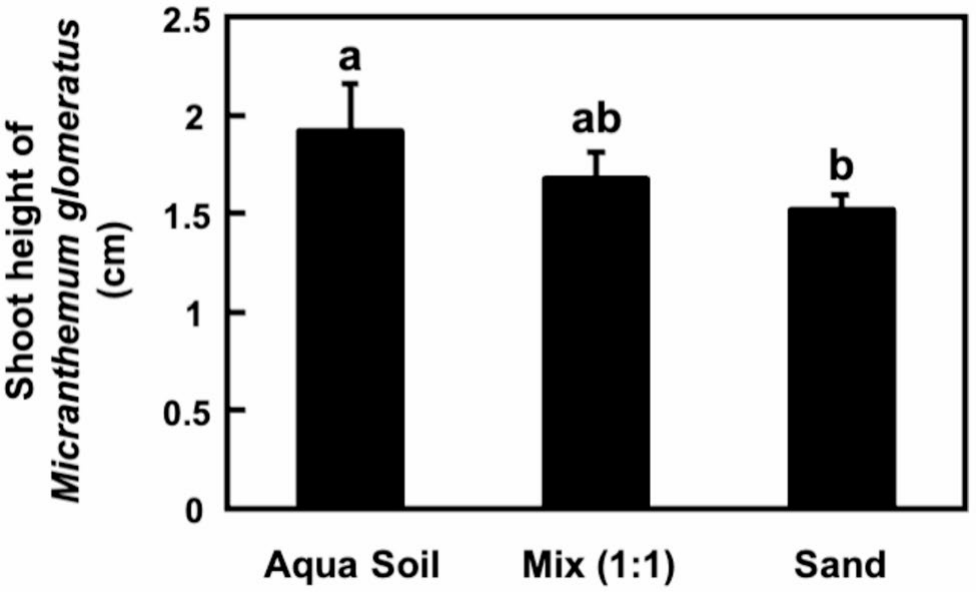
Aerial part height of *Micranthemumglomeratus* after ex vitro transplantation into different substrates for 4 weeks. Data are presented as mean ± standard deviation (*n* = 4). Distinct symbols indicate significant differences between groups (Duncan's new multiple range test, *p* < 0.05)

We next conducted comparative analysis of the influence of different substrate formulations on the acclimatization of *Micranthemum* sp. ‘Monte Carlo’ (Fig. 4) and *M. glomeratus* (Fig. 5) following in vitro culture. The experiment demonstrated a 100% survival rate for the transplanted plantlets across all tested substrates: Silica sand, a 1:1 mixture of aqua soil and silica sand, and aqua soil exclusively. Observations on growth dynamics revealed that *Micranthemum* sp. ‘Monte Carlo’ achieved optimal growth in pure aqua soil, with the mixed substrate as the second most favorable environment, while performance in silica sand was marginally less satisfactory. In parallel, the growth pattern of *M. glomeratus*, which typically exhibits an upright growth orientation, similarly favored aqua soil as evidenced by plant height measurements, with secondary preference given to the mixed substrate, and relatively lower growth rates in silica sand (Fig. 6).

**Fig. 7:**
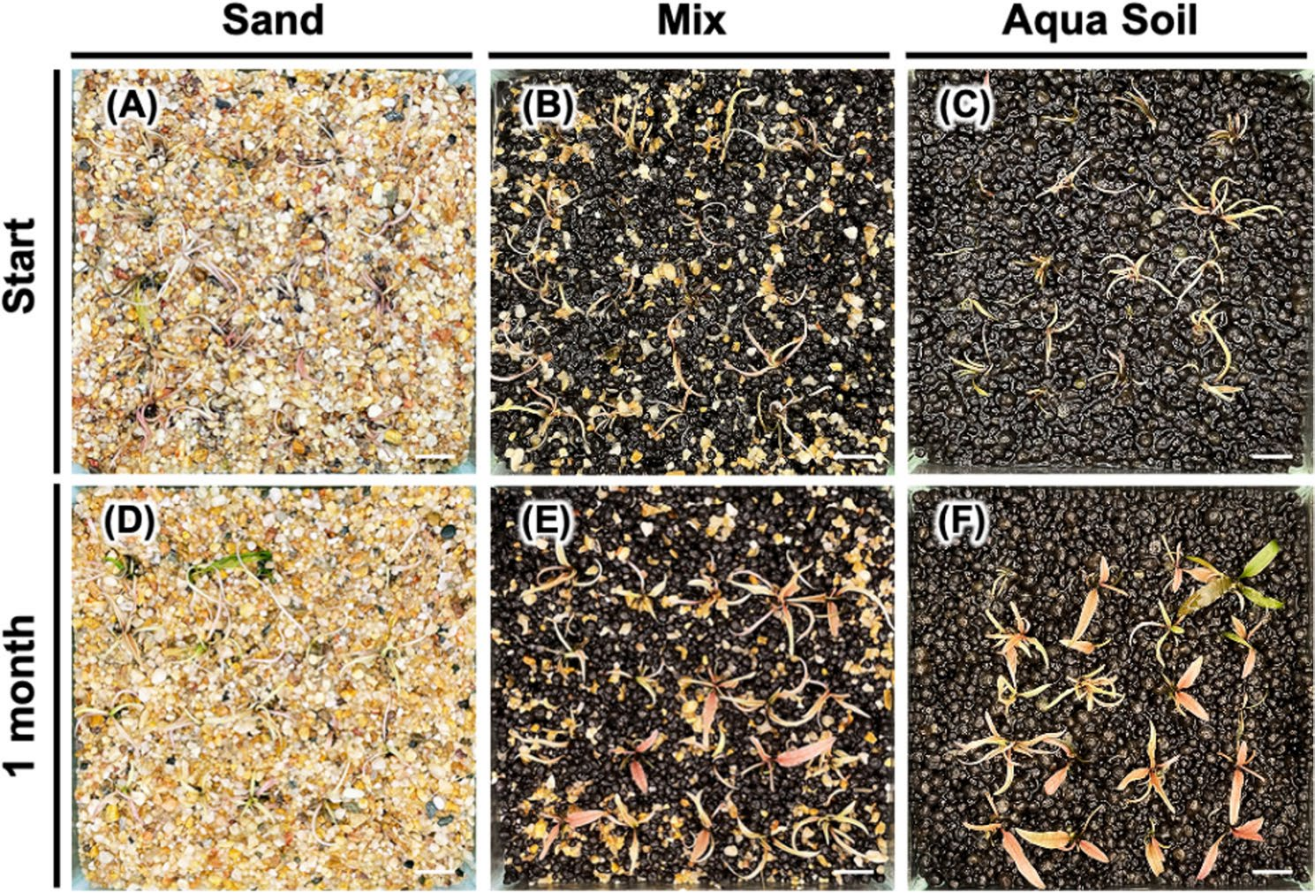
Impact of substrate combinations on ex vitro transplanted *Cryptocoryne* sp.‘Flamingo’. Plants were transplanted into US fine sand (**A**), mixed substrate (**B**), and black soil (**C**) and cultivated for one month (**D**, **E**, **F**)

The growth for *Cryptocoryne* sp. ‘Flamingo’ exhibited similar patterns observed with *Micranthemum* sp. ‘Monte Carlo’ and *M. glomeratus*, showing a 100% survival rate irrespective of the substrate used (Fig. 7). Notably, the growth performance was superior in aqua soil and the mixed substrate, evidenced by the proliferation of foliage, in contrast to the markedly diminished growth observed in silica sand substrate.

## Discussion

This study tackled the challenges associated with nematode infections in aquatic ornamental plants and investigated the in vitro propagation of *Micranthemum* sp. ‘Monte Carlo’, *M. glomeratus*, and *Cryptocoryne* sp. ‘Flamingo’. The research identified key factors for successful tissue culture propagation, including the efficacy of plant growth regulators and optimization of substrates for plantlet acclimatization. The findings offer valuable knowledge for improving the tissue culture propagation of aquatic plants, contributing to both sustainable horticulture and management of nematode infections.

Effective sterilization protocol is essential for the establishment of phytopathogen and nematode-free tissue cultures. Our results showed that 0.5% sodium hypochlorite solution for 30 min was the most effective in reducing contamination while maintaining explant viability. Higher concentrations or extended soaking times led to tissue damage and whitening, consistent with prior studies in *Micranthemum callitrichoides* ‘Cuba’ [7], where higher concentrations (5%) caused significant explant mortality. This optimized protocol ensures the viability of explants, while adequately removing pathogens, thereby enhancing subsequent in vitro development.

The growth responses of *Micranthemum* sp. ‘Monte Carlo’ and *M. glomeratus* in PGR-free media highlighted that omitting PGRs resulted in the largest clump diameters and clear nodal organization. In contrast, increasing PGR concentrations, particularly BAP, negatively impacted shoot elongation and clump size. Similar inhibitory effects of PGRs on shoot proliferation in *Hemianthus callitrichoides* have been reported [9].

In this study, *Cryptocoryne* sp. ‘Flamingo’ responded positively to increased cytokinin concentrations, especially in combination with NAA, which enhanced the differentiation of adventitious buds. This observation is consistent with previous researches on *Cryptocoryne* spp., showing that higher levels of BAP, in combination with NAA, can increase the number of buds [16, 20, 21]. Such evidence supports the premise that a higher cytokinin-to-auxin ratio is conducive to bud proliferation [27, 28]. The optimal bud proliferation for *Cryptocoryne* sp. ‘Flamingo’ was achieved with a combination of BAP (4.0 and 6.0 mg/L) and NAA (0.1 mg/L), highlighting the importance of applying BAP in the propagation of adventitious buds in this species.

Interestingly, root formation in *Micranthemum* sp. was impeded by the presence of PGRs, which is consistent with previous findings [7–9], suggesting that PGR-free media foster successful root development. This phenomenon suggests the heightened sensitivity of both *Micranthemum* sp. ‘Monte Carlo’ and *M. glomeratus* to 6-BA, where its presence significantly suppresses root differentiation. This aligns with previous findings, which suggest that BAP impedes the regeneration of roots in plants [29, 30], Consequently, the optimal condition for root induction in these aquatic plants appears to be the absence of any plant growth regulators. Conversely, *Cryptocoryne* sp. ‘Flamingo’ displayed distinct response, where BAP did not suppress root differentiation, highlighting species-specific hormonal response [31–34]. The systematic examination of PGR effects on bud proliferation revealed a pronounced influence of 6-BA, particularly when combined with NAA, significantly promoted adventitious bud formation. This highlights the complex and variable interactions between PGRs and root development in aquatic plants. While elevated cytokinin levels from adventitious bud proliferation often hinder root induction [33], the results this study demonstrate the need for tailored PGR applications or absence thereof to effectively promote root development in different plant species.

The acclimatization phase is a critical juncture for ensuring the survival and growth of tissue-cultured plantlets when transferred to ex vitro conditions. This transition can pose challenges due to fluctuations in humidity levels, which may result in seedling mortality. However, the leaf morphology of amphibious plants exhibit remarkable adaptability to aquatic environments, including significant reductions in stomatal number and cuticle thickness—adaptations that are well-suited for submerged conditions [35].

This inherent trait facilitates their direct transplantation into aquatic settings without the need for an elaborate acclimatization regimen, thereby significantly mitigating loss rates. The efficiency of this acclimatization process without substantial loss has been previously documented for miniature pearlweed (*Micranthemum callitrichoides* ‘Cuba’) and Wendt’s water trumpet (*Cryptocoryne wendtii*) [7, 20]. Based on the findings of this study, aqua soil emerges as the preferred substrate for the acclimatization of these aquatic plant seedlings, promoting robust growth post-tissue culture. For those using silica sand as the primary substrate, incorporating base fertilizer is advisable to enhance nutrient availability and support healthy development of the plantlets.

In conclusion, this study provides a comprehensive framework for the in vitro propagation and acclimation of ornamental aquatic plants. The optimized sterilization, PGR, and substrate conditions facilitate large-scale propagation, which is essential for meeting the demands of the ornamental plant industry while ensuring pathogen-free plant material. The findings also contribute to sustainable cultivation practices by mitigating the challenges posed by nematode infestations. cultivation strategies, thereby contributing to the preservation of aquatic biodiversity.

## Data Availability

All data supporting the findings of this study are available within the paper.

## References

[1] Maki K, Galatowitsch S. Movement of invasive aquatic plants into Minnesota (USA) through horticultural trade. Biol Conserv. 2004;118:389–96.

[2] Karimi Alavijeh M, Safi S, Zarei A. An efficient method for economic micropropagation of three aquatic plant species (*Lobelia cardinalis*, *Staurogyne repens*, and *Alternanthera reineckii*). Aquac Int. 2023;31:1623–36.

[3] Mansour AT, Ashour M, Alprol AE, Alsaqufi AS. Aquatic plants and aquatic animals in the context of sustainability: cultivation techniques, integration, and blue revolution. Sustainability. 2022;14:3257.

[4] Hiscock P. Encyclopedia of aquarium plants. New York: Barron’s Educational Series, Inc. Hauppauge; 2003.

[5] Acevedo-Rodríguez P, Strong MT. Catalogue of seed plants of the West Indies. Smithson Contrib Bot. 2012;98:1–1192.

[6] Govaerts R, Nic Lughadha E, Black N, Turner R, Paton A. The world checklist of vascular plants, a continuously updated resource for exploring global plant diversity. Sci Data. 2021;8:215. 34389730 10.1038/s41597-021-00997-6 PMC8363670

[7] Barpete S, Özcan SF, Aasim M, Özcan S. *In vitro* high frequency regeneration through apical shoot proliferation of *Hemianthus callitrichoides* ‘Cuba’-a multipurpose ornamental aquatic plant. Turk J Biol. 2015;39:493–500.

[8] Ing NS, Kharuddin AA, Sahidin N, Zainuddin R, Mahmud NH, Abdullah TA, Ha HC. *Vitro* micropropagation of aquarium plants Pearl grass *Hemianthus micranthemoides* (Nuttall) and micro sword grass *Lilaeopsis Brasiliensis* (Glaziou) affolter (Apiaceae). J Agrobiotech. 2019;10:88–93.

[9] Özcan E, Atar HH, Ali SA, Aasim M. Artificial neural network and decision tree–based models for prediction and validation of *in vitro* organogenesis of two hydrophytes—*Hemianthus callitrichoides* and *Riccia fluitans*. Vitro Cell Dev Biol Plant. 2023;59:547–62.

[10] Burkill IH. A dictionary of the economic products of the Malay Peninsula, A Dictionary of the Economic Products of the Malay Peninsula. 2nd ed. 1966. Vol. 1–2.

[11] Dötsch A. *Cryptocoryne wendtii* de Wit (Pflanzenportrait). Aqua Planta. 1984;84:17–8.

[12] Kane M, Davis G, Hoffner T, Henny R. Gibberellins promote flowering in two *Cryptocoryne* species. HortScience. 1995;30:380.

[13] Kane ME, Davis GL, McConnell DB, Gargiulo JA. *In vitro* propagation of *Cryptocoryne wendtii*. Aquat Bot. 1999;63:197–202.

[14] Kane ME, Gilman EF, Jenks MA, Sheehan TJ. Micropropagation of the aquatic plant *Cryptocoryne Lucens*. HortScience. 1990;25:687–9.

[15] Rosen DJ. *Cryptocoryne beckettii* (Araceae), a new aquatic plant in Texas. Bot Res Inst Tex. 2000;19:399–401.

[16] Stanly C, Bhatt A, Keng CL. An efficient *in vitro* plantlet regeneration of C*ryptocoryne wendtii* and *Cryptocoryne Becketti* through shoot tip culture. Acta Physiol Plant. 2011;33:619–24.

[17] Bambaranda B, Peiris S. *Cryptocoryne wendtii* can successfully be grown in river sand enriched with nutrients. Sri Lanka J Aquat Sci. 2016;21:67–71.

[18] Ahmad A, Ismun A, Taib M, Ilias MA, Ismail A, Othman R, Zainudin S. Effects of salinity stress on carbohydrate metabolism in *Cryptocoryne elliptica* cultures. J Trop Plant Physiol. 2017;9:1–13.

[19] Dissanayake C, Hettiarachchi M, Iqbal M. Sustainable use of *Cryptocoryne wendtii* and *Echinodorus cordifolius* in the aquaculture industry of Sri Lanka by micropropagation. Sri Lanka J Aquat Sci. 2007;12:89–101.

[20] Unal S, Turkmen G, Yagmur B, Bayraktar M, Gurel A. Improved *in vitro* propagation and direct acclimatization of *Cryptocoryne wendtii* in aquarium in the presence of aquarium fish *Puntius tetrazona* (Bleeker). Indian J Exp Biol. 2019;57:330–7.

[21] Klaocheed S, Jehsu W, Choojun W, Thammasiri K, Prasertsongskun S, Rittirat S. Induction of direct shoot organogenesis from shoot tip explants of an ornamental aquatic plant, *Cryptocoryne wendtii*. Walailak J Sci Tech. 2020;17:293–302.

[22] Hung S, Tsay T, Yen J, Chen P. The survey of nematodes on the commercial submerged aquatic plants in Taiwan. Plant Pathol Bull. 2015;24:37–52.

[23] Khamushi M, Dehestani-Ardakani M, Zarei A, Kamali Aliabad K. An efficient protocol for micropropagation of old Cypress of Abarkuh (*Cupressus sempervirens* var. Horizontalis [Mill.]) under *in vitro* condition. Plant Cell Tiss Organ Cult. 2019;138:597–601.

[24] Ozcan E, Onlu S, Sezgin ME, Barpete S. The effect of improvised media and sugar concentration on *in vitro* shoot multiplication of *Riccia fluitans* L.: an amphibious liverwort. Fresenius Environ Bull. 2021;30:1696–702.

[25] Alavijeh MK, Ebadi A, Zarei A, Omidi M. Somatic embryogenesis from anther, whole flower, and leaf explants of some grapevine cultivars. Plant Tissue Cul Biotechnol. 2016;26:219–30.

[26] Murashige T, Skoog F. A revised medium for rapid growth and bio assays with tobacco tissue cultures. Physiol Plant. 1962;15:473–97.

[27] Haque SM, Ghosh B. A submerged culture system for rapid micropropagation of the commercially important aquarium plant,‘amazon sword’(*Echinodorus* ‘Indian Red’). Vitro Cell Dev Biol Plant. 2019;55:81–7.

[28] Jabir T, George S, Raj A, Lakshmi S, Joseph A. Micropropagation and *in vitro* flowering of an ornamental aquarium plant *Lindernia antipoda* (L.) Alston. Intl J Aquac. 2016;6:1–10.

[29] Malá J, Máchová P, Cvrčková H, Karady M, Novák O, Mikulík J, Hauserová E, Greplová J, Strnad M, Doležal K. Micropropagation of wild service tree (*Sorbus torminalis* [L.] Crantz): the regulative role of different aromatic cytokinins during organogenesis. J Plant Growth Regul. 2009;28:341–8.

[30] Kanchanapoom K, Chunui P, Kanchanapoom K. Micropropagation of *Anubias Barteri* var. Nana from shoot tip culture and the analysis of ploidy stability. Not Bot Horti Agrobot Cluj-Napoca. 2012;40:148–51.

[31] Fasolo F, Zimmerman RH, Fordham I. Adventitions shoot formation on excised leaves of *in vitro* grown shoots of Apple cultivars. Plant Cell Tiss Organ Cult. 1989;16:75–87.

[32] Öztürk M, Khawar KM, Atar HH, Sancak C, Ozcan S. *In vitro* micropropagation of the aquarium plant *Ludwigia repens*. Asia Pac J Mol Biol Biotechnol. 2004;12:21–5.

[33] Huetteman CA, Preece JE. Thidiazuron: a potent cytokinin for Woody plant tissue culture. Plant Cell Tiss Organ Cult. 1993;33:105–19.

[34] Yusnita S, Geneve R, Kester S. Micropropagation of white flowering Eastern Redbud (*Cercis Canadensis* var. Alba L). J Environ Hortic. 1990;8:177–9.

[35] Koga H, Ikematsu S, Kimura S. Diving into the water. Amphibious plants as a model for investigating plant adaptations to aquatic environments. Annu Rev Plant Biol. 2024;75:579–604. 38424069 10.1146/annurev-arplant-062923-024919

